# IRE1α Is Associated with Host Lipid Biosynthesis During Human Coronavirus OC43 Infection

**DOI:** 10.3390/v18090996

**Published:** 2026-09-10

**Authors:** Kaitlin N. James, Jennifer A. Miller, Hanna C. Huston, Jessica M. Oda, Susan L. Fink

**Affiliations:** 1 Department of Laboratory Medicine and Pathology, University of Washington, Seattle, WA 98195, USA; 2 Interdisciplinary Graduate Program in Immunology, University of Iowa, Iowa City, IA 52242, USA; 3 Department of Emergency Medicine, University of Iowa, Iowa City, IA 52242, USA

**Keywords:** coronavirus, IRE1α, ER stress, unfolded protein response, lipid, metabolism

## Abstract

Research into the replication of human coronaviruses has surged, but much remains unknown regarding the host factors that influence disease. These viruses often activate cellular endoplasmic reticulum (ER) stress responses, benefiting from incompletely understood downstream effects. In this study, we investigated the role of the host ER stress response component, inositol-requiring enzyme 1 α (IRE1α), in regulating lipid metabolism during human coronavirus OC43 (HCoV-OC43) infection. We found IRE1α-dependent induction of lipogenesis-related genes, increased metabolic intermediates, and enhanced lipophilic membrane staining during infection. Inhibition of de novo fatty acid synthesis impaired HCoV-OC43 replication, while bypassing the first steps in de novo lipogenesis with supplementation of exogenous palmitate restored viral replication in the setting of IRE1α inhibition. Together, these results suggest that lipid metabolic remodeling is associated with IRE1α signaling during coronavirus infection and targeting these host pathways may represent a potential antiviral strategy.

## 1. Introduction

Human coronaviruses are enveloped positive-sense RNA viruses that cause respiratory infections ranging in severity from mild, self-limited upper respiratory symptoms to severe pneumonia with acute respiratory distress syndrome, multiorgan failure, and the potential for chronic post-infectious sequelae [1]. Endemic coronaviruses such as HCoV-OC43 contribute substantially to seasonal respiratory illness, while highly pathogenic emergent coronaviruses including SARS-CoV, MERS-CoV, and SARS-CoV-2 have caused significant fatalities, particularly among those who have one or more pre-existing conditions. Coronaviruses manipulate host cellular processes to facilitate their replication; therefore, a better understanding of host factors that promote infection may inform strategies to protect at-risk patients.

Clinical studies during the COVID-19 pandemic established diabetes, advanced age, hypertension, chronic kidney disease, and obesity as risk factors for severe SARS-CoV-2 infection [2,3,4]. Although the mechanisms contributing to disease severity are likely multifactorial, these risk factors are also associated with endoplasmic reticulum (ER) stress [5,6,7]. ER stress occurs when the protein folding and processing capacity of the ER is overwhelmed, triggering the cellular unfolded protein response to activate compensatory pathways. The unfolded protein response component IRE1α is an ER-resident transmembrane protein that senses ER stress, which activates its cytosolic RNase domain. Activated IRE1α initiates nonconventional splicing of *XBP1* mRNA. Spliced XBP1 encodes a transcription factor, which induces condition-specific upregulation of genes involved in ER function, protein synthesis, and metabolism [8,9,10].

Several studies have demonstrated that human coronaviruses activate IRE1α, which promotes efficient viral replication [11,12,13,14]. In addition to the ER stress response initiated by viral infection, multiple lines of evidence suggest that exogenous IRE1α activation may further augment coronavirus replication [11,12]. There may be multiple mechanisms by which IRE1α promotes coronavirus infection, including elevation of host NUAK2, which promotes spike-dependent coronavirus entry [12]. IRE1α may also promote post-entry viral RNA replication [11], although the underlying mechanisms remain unknown.

Increasing evidence demonstrates that coronaviruses manipulate host lipid biosynthetic pathways to promote efficient replication [15,16], although the upstream signaling mechanisms coordinating these metabolic changes are not well-understood. Given the role of IRE1α and XBP1 in promoting lipogenesis, we hypothesized that IRE1α may support coronavirus replication through regulation of de novo fatty acid synthesis. Here we identify lipid metabolic remodeling as a potential link associated with IRE1α signaling during human coronavirus infection.

## 2. Materials and Methods

### 2.1. Reagents

Cells were pretreated for 2.5 h with 25–50 µM 4μ8C (8-formyl-7-hydroxy-4-methylcoumarin, MedChemExpress, Monmouth Junction, NJ, USA), 50 µM AMC (7-amino-4-methylcoumarin, VWR, Radnor, PA, USA), 10 µM KIRA8 (MedChemExpress, Monmouth Junction, NJ, USA), 25–100 µM CP-640186 (MedChemExpress, Monmouth Junction, NJ, USA), or matched concentrations of DMSO vehicle control (Fisher Scientific, Waltham, MA, USA). Cells were treated with 100 µM BSA-Palmitate Saturated Fatty Acid Complex (Cayman Chemical, Ann Arbor, MI, USA) or 100 µM BSA Control for BSA-Fatty Acid Complexes (Cayman Chemical, Ann Arbor, MI, USA) at the time of HCoV-OC43 infection. Cells were treated with 10 µM IXA4 (Cayman Chemical, Ann Arbor, MI, USA) for 24 h. Viability was assessed by quantifying ATP in metabolically active cells using the CellTiter-Glo 2.0 Assay (Promega, Madison, WI, USA).

### 2.2. Cells and Viruses

Cells were propagated in Dulbecco’s modified Eagle’s medium (DMEM) (Gibco, Waltham, MA, USA) supplemented with 10% fetal bovine serum (FBS) (Serum Plus II, MilliporeSigma, St. Louis, MO, USA), 10 mM HEPES (Gibco, Waltham, MA, USA), 50 U/mL penicillin–streptomycin (Gibco, Waltham, MA, USA), and 0.05 mM β-mercaptoethanol (MilliporeSigma, Burlington, MA, USA). HCT-8 cells were obtained from the American Type Culture Collection (CCL-244). A549 cells expressing Human Angiotensin-Converting Enzyme 2 (ACE2) (NR-53821) and HCoV-OC43 (NR-52725) were obtained through BEI Resources, National Institute of Allergy and Infectious Diseases (NIAID), National Institutes of Health (NIH). HCoV-OC43 was propagated in HCT-8 cells using medium containing 2% FBS. HCoV-OC43 was titered in HCT-8 cells using a focus-forming assay and used for infections at an MOI of 0.01 for 48 h.

### 2.3. Expression Analysis

RNA isolated from cell lysates using the SingleShot Cell Lysis Kit (Bio-Rad, Hercules, CA, USA) was used to synthesize complementary DNA (cDNA) using the iScript cDNA Synthesis Kit (Bio-Rad, Hercules, CA, USA). Quantitative RT-PCR (qRT-PCR) was performed on a Bio-Rad CFX Duet using SYBR Green (Bio-Rad, Hercules, CA, USA) with the following primers (all primers listed in the 5′–3′ orientation): human spliced *XBP1*: CTG AGT CCG CAG CAG GTG (forward) and TGC CCA ACA GGA TAT CAG ACT (reverse); human *ERDJ4*: TAG TCG GAG GGT GCA GGA TA (forward) and CGC TCT GAT GCC GAT TTT GG (reverse); HCoV-OC43 *M*: ATG TTA GGC CGA TAA TTG AGG ACT AT (forward) and AAT GTA AAG ATG GCC GCG TAT T (reverse); HCoV-OC43 *ORF1ab*: TGG ATT TTG GCG GGA TGG AA (forward) and GAG ACG GGC ATC TAC ACT CG (reverse); human *ACACB*: GGG TCA TCG AGA AGG TGC TT (forward) and GGG GGT CAC CAT CAC AAC AA (reverse); human *SCD1*: TGC GAT ATG CTG TGG TGC TT (forward) and TTG TGG AAG CCC TCA CCC AC (reverse); human *FASN*: GTC TTG AAC TCC TTG GCG GA (forward) and AGG AAG ATA GCC ATG CCG AG (reverse); human *RPS18*: GGG TGT GGG CCG AAG ATA TG (forward) and TGA TCA CAC GTT CCA CCT CA (reverse).

Melt curve analysis was used to confirm that single reaction products were produced, and expression was calculated relative to the housekeeping gene *RPS18*.

### 2.4. Malonyl-CoA Quantification

HCT-8 cells were pretreated with acetyl-CoA carboxylase (ACC) inhibitor CP-640186 2.5 h ahead of HCoV-OC43 infection at an MOI of 0.01. At 48 h post-infection, cells were harvested using 0.05% trypsin solution containing 2 mM EDTA, pelleted by centrifugation and resuspended in diluent. A tissue homogenizer was used to lyse cells, and lysates were cleared by centrifugation. Total protein concentration was measured using a biospectrophotometer (DeNovix, Wilmington, DE, USA) and malonyl-CoA was quantified by ELISA (CUSABIO, Houston, TX, USA) per the manufacturer’s recommendations, with optical density measured using a SpectraMax plate reader (Molecular Devices, San Jose, CA, USA). Malonyl-CoA concentration is reported as pmol/mg based on a standard curve generated using materials supplied in the ELISA kit.

### 2.5. Fluorescence Microscopy and Analysis

A549 cells expressing ACE2 were fixed with 2% paraformaldehyde and permeabilized with 0.1% TritonX-100 in PBS. Biotium’s fluorescent CellBrite Blue Cytoplasmic Membrane Stain (#30024, Fremont, CA, USA) was used per the manufacturer’s recommendation for fixed cells to visualize polar lipids. Images were taken across two replicate wells per condition using the DAPI fluorescent channel at 10× magnification using a BioTek Cytation 1 imaging reader (Fisher Scientific, Palatine, IL, USA, Gen5 version 3.17). ImageJ (version 1.46r) was used to analyze the images by defining cells and generating a Mean Gray Value on a per-cell basis to quantify the fluorescence intensity.

### 2.6. RNA Sequencing Analysis

Previously published [12] RNA sequencing data (ENA PRJEB51271) were reanalyzed to identify IRE1α-dependent regulation of lipogenesis-related genes during SARS-CoV-2 infection. Preprocessed differential expression (DESeq2 v1.28.1, R v4.0.0) tables from the authors’ supplementary GitHub repository (boutroslab/Supp_Prasad_2022) were used for secondary reanalysis in this study. The provided DESeq2 output tables were analyzed to determine IRE1α-dependent lipogenesis genes for this study. Infection-associated transcript changes were identified by comparing SARS-CoV-2-infected cells to non-infected cells with DESeq2. IRE1α-inhibition-associated changes during infection were defined by comparing infected cells treated with 100 µM 4µ8C or DMSO as a control. We defined a lipogenesis gene set by combining MSigDB (release v2025.1.Hs) Reactome pathways containing lipogenesis or triglyceride biosynthesis terms, Gene Ontology biological process terms related to triglyceride/lipid biosynthesis, and a selected set of core lipogenic regulators and enzymes. Genes were classified as IRE1α-dependent within the lipogenesis gene set if they showed a log2 fold change > 0.5 in the infection comparison and log2 fold change < −0.5 in the 4µ8C versus DMSO comparison. Lipogenesis genes meeting these criteria were plotted as log2 fold change (SARS-CoV-2 vs. mock uninfected) versus log2 fold change (4µ8C vs. DMSO, infected). Threshold lines at ±0.5 log2 fold change are shown as dotted lines. All secondary analyses and visualizations were performed in R v4.4.2 using msigdbr v 25.1.1, dplyr v1.1.4, data.table v1.18.0, ggplot2 v4.0.1, and ggrepel v0.9.6.

### 2.7. Statistics and Reproducibility

ANOVA was used for comparisons between two or more groups. Multiple comparisons were controlled for by using Šídák’s or Dunn’s multiple comparisons test. *p* values of less than 0.05 were considered statistically significant.

## 3. Results

### 3.1. IRE1α Promotes Expression of Enzymes Involved in Fatty Acid Synthesis During HCoV-OC43 Infection

**Figure 1:**
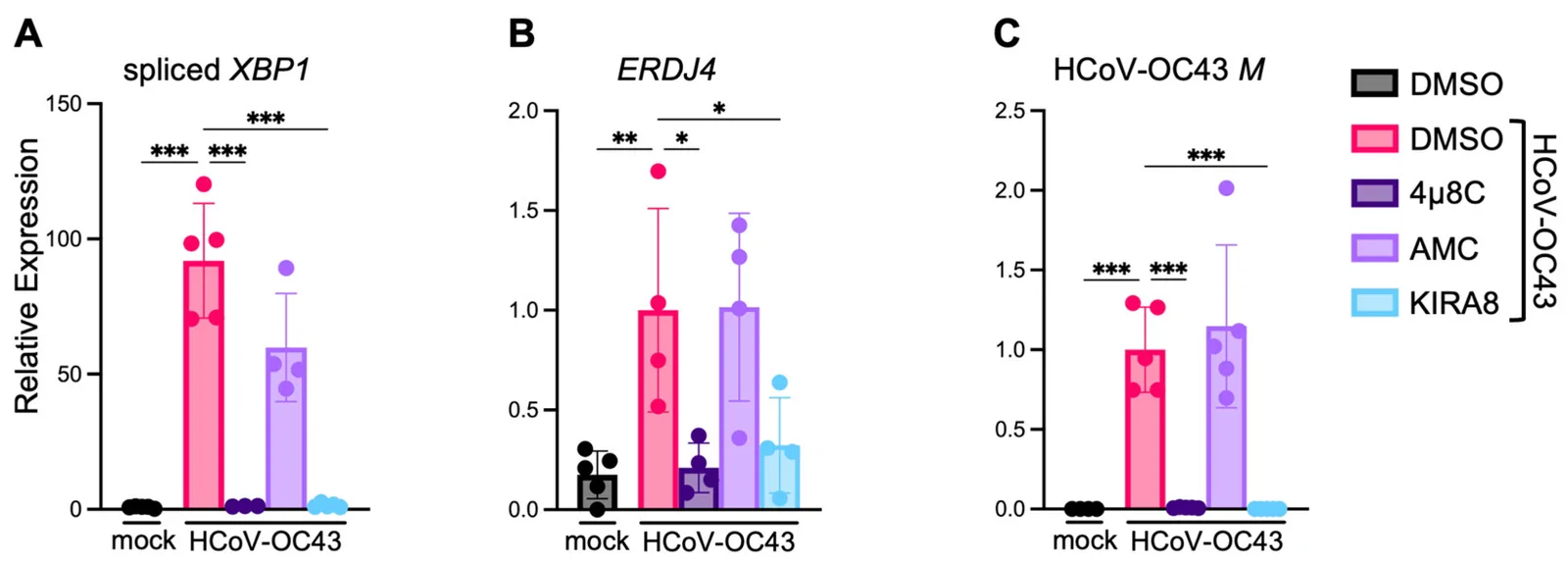
Activation of IRE1α promotes HCoV-OC43 infection. (**A**–**C**) Cells were pretreated with DMSO vehicle control, IRE1α nuclease inhibitor 4µ8C, inactive control AMC, or IRE1α kinase and nuclease inhibitor KIRA8. Treated cells were infected with HCoV-OC43 at an MOI of 0.01 and RNA was harvested 48 h post-infection for quantitative RT-PCR analysis. Relative expression was determined for spliced *XBP1* (**A**), *ERDJ4* (**B**), HCoV-OC43 viral RNA (**C**). Data are means +/− SD from five replicate wells of a cell culture plate and are representative of three independent experiments. * *p* < 0.05, ** *p* < 0.01, *** *p* < 0.001 by one-way ANOVA.

We previously found that HCoV-OC43 infection activates IRE1α in human HCT-8 epithelial cells [11]. To determine whether this result extends to human lung epithelial cells, we infected A549 cells with HCoV-OC43 for 48 h and found robust induction of spliced *XBP1* (Figure 1A). The IRE1α nuclease inhibitor, 4μ8C [17], but not the structurally similar inactive control molecule AMC [18,19], prevented *XBP1* mRNA splicing (Figure 1A), without evidence of cytotoxicity (Figure S1), confirming the requirement for IRE1α enzymatic activity. In addition, a structurally distinct, selective IRE1α nuclease inhibitor, KIRA8 [20], also prevented *XBP1* mRNA splicing in HCoV-OC43 infected cells (Figure 1A). The protein product of spliced *XBP1* mRNA encodes a transcription factor that upregulates targets involved in ER function, including *ERDJ4* [10]. We found an IRE1α-dependent increase in *ERDJ4* expression during HCoV-OC43 infection (Figure 1B), indicating expression of XBP1-dependent genes. We then quantified viral RNA by qRT-PCR and observed a substantial reduction with the IRE1α inhibitor 4μ8C, but not with the inactive control AMC (Figure 1C). The distinct IRE1α nuclease inhibitor, KIRA8 similarly decreased the abundance of viral RNA (Figure 1C), demonstrating that the role of IRE1α to support efficient HCoV-OC43 infection in HCT-8 cells [11] extends to human lung cells.

The IRE1α-XBP1 pathway drives transcriptional changes affecting multiple aspects of cellular physiology. Prior RNA sequencing from SARS-CoV-2 infection in the presence or absence of the IRE1α inhibitor 4μ8C revealed gene set enrichment of pathways including fatty acid metabolism [12]. Our additional analysis using the raw RNA sequencing results from this study demonstrated IRE1α-dependent expression of multiple genes in lipogenesis-related categories (Figure S2A). Fatty acid synthesis is driven by a coordinated network of enzymes that regulate the production and modification of lipids [21]. Acetyl-CoA is converted to malonyl-CoA by acetyl-CoA carboxylase (ACC), the rate-limiting enzyme in de novo lipogenesis. Fatty acid synthase (FASN) then catalyzes the synthesis of long-chain saturated fatty acids, primarily palmitate. Stearoyl-CoA desaturase 1 (SCD1) subsequently converts saturated fatty acids into monounsaturated fatty acids that are essential for membrane biosynthesis, energy storage, and cellular signaling. We therefore assessed expression of the genes encoding these enzymes in HCoV-OC43-infected cells (Figure S2B–D). We found that the IRE1α inhibitor 4μ8C reduced mRNA abundance of *ACACB* (encoding ACC2), *SCD1*, and *FASN* in HCoV-OC43-infected cells (Figure S2B–D). Together, these results suggest that IRE1α promotes expression of genes related to fatty acid synthesis during human coronavirus infection.

### 3.2. Fatty Acid Synthesis Metabolic Intermediates Are Increased During HCoV-OC43 Infection and Are Required for Viral Replication

**Figure 2:**
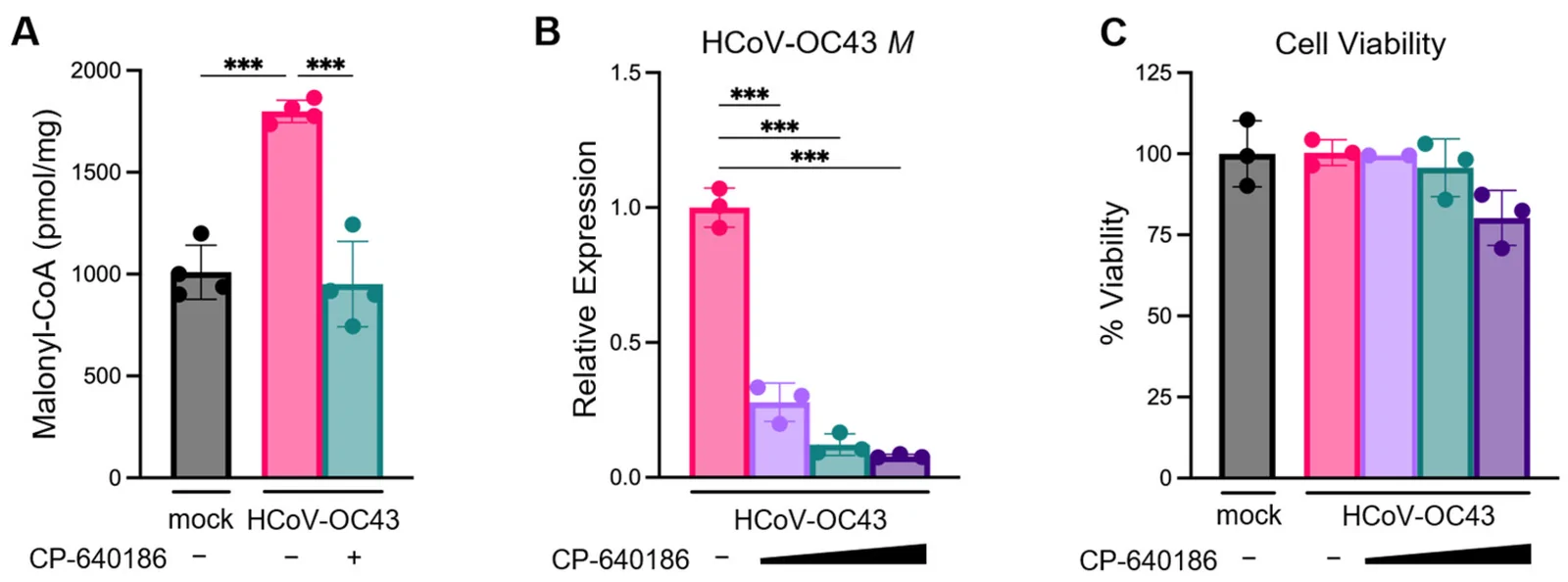
Fatty acid synthesis metabolic intermediates are increased during HCoV-OC43 infection and are required for viral replication. (**A**) Cells were pretreated with ACC inhibitor CP-640186, then infected with HCoV-OC43 at an MOI of 0.01. Cells were harvested and lysed 48 h post-infection. Malonyl-CoA was quantified using an ELISA and normalized to total protein content. Data are means +/− SD from four technical replicates and are representative of three independent experiments. (**B** + **C**) Cells were pretreated with ACC inhibitor CP-640186 and then infected with HCoV-OC43. RNA was harvested from samples 48 h following infection for quantitative RT-PCR analysis. Relative expression was determined for HCoV-OC43 viral RNA (**B**). Samples were assessed for cell viability using CellTiter-Glo (**C**). Data are means +/− SD from three replicate wells of a cell culture plate and are representative of three independent experiments. *** *p* < 0.001 by one-way ANOVA.

We next sought to determine whether these transcriptional changes produced functional metabolic remodeling by assessing a key intermediate in the lipogenesis pathway. Therefore, we quantified malonyl-CoA, which is generated by the rate-limiting enzyme ACC. We found increased malonyl-CoA in HCT-8 cells infected with HCoV-OC43, compared to mock uninfected controls (Figure 2A). We further observed that malonyl-CoA was reduced in infected cells by the selective ACC inhibitor CP-640186 [22] (Figure 2A), consistent with the established role of ACC in malonyl-CoA production.

We next investigated the effects of ACC inhibition during infection with HCoV-OC43. Increasing concentrations of CP-640186 yielded a dose-dependent reduction in viral replication, as assessed by measuring viral RNA with qRT-PCR (Figure 2B). Viral RNA was reduced by concentrations of CP-640186 that had no effect on cell viability (Figure 2C), indicating that the effect of this inhibitor was not due to cytotoxicity. Together, these results suggest a requirement for ACC enzymatic activity and de novo lipogenesis for optimal HCoV-OC43 infection.

### 3.3. IRE1α-Dependent Increase in Lipid-Associated Membrane Staining During HCoV-OC43 Infection

**Figure 3:**
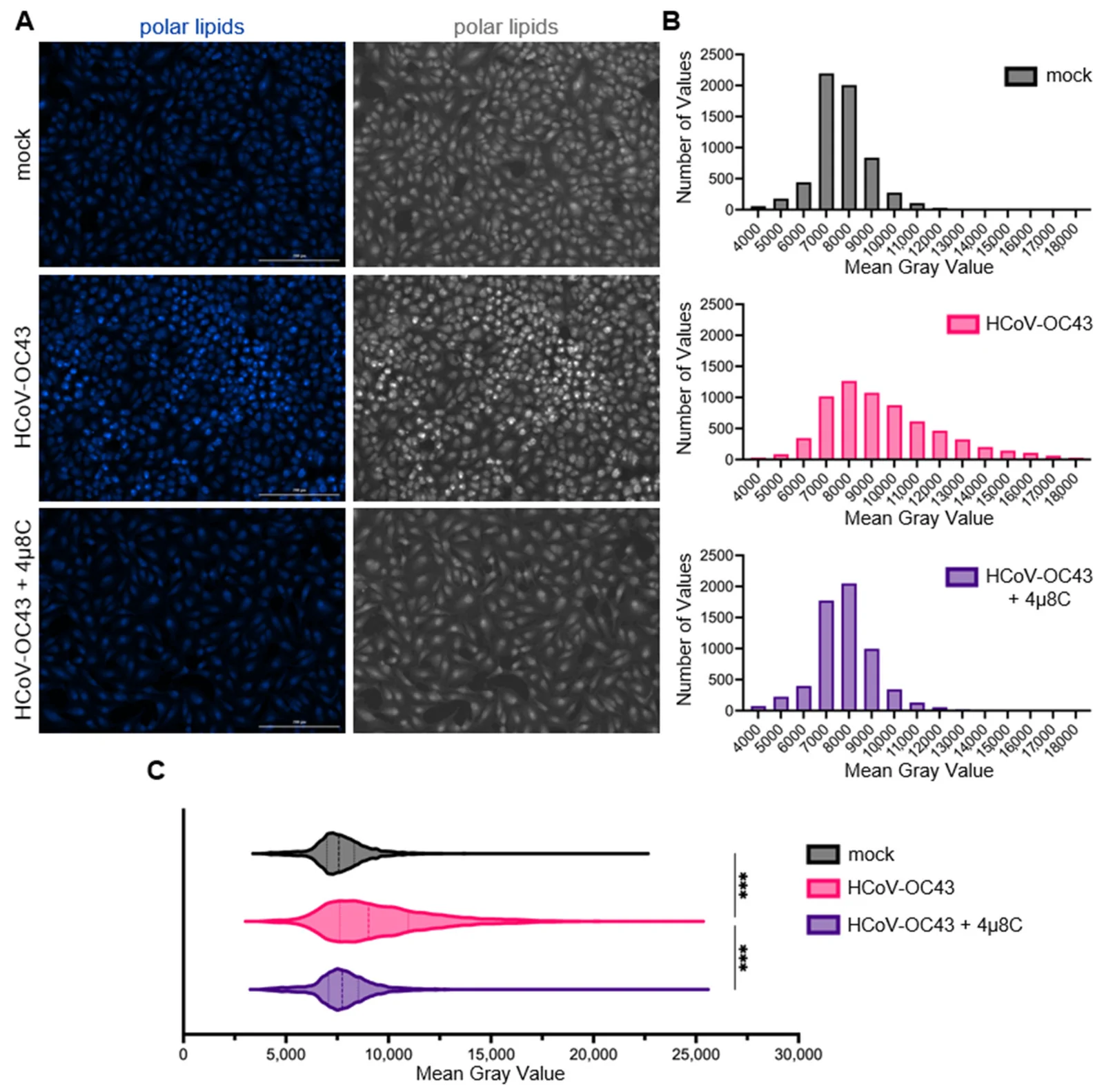
IRE1α-dependent increase in lipid stain intensity during HCoV-OC43 infection. (**A**–**C**) Cells were pretreated with DMSO vehicle control or IRE1α inhibitor 4µ8C, then infected with HCoV-OC43 for 48 h. Staining was performed on fixed cells for polar lipids (blue in colored images). Scale bar equals 200 µm. Fluorescence intensity of each cell visualized was quantified as a Mean Gray Value (**B** + **C**). Sixteen images were captured over two replicate wells, per condition, and are representative of three biologically independent experiments. *** *p* < 0.001 by one-way ANOVA computed using Mean Gray Values from individual cells.

Malonyl-CoA produced by ACC is subsequently consumed by FASN, which catalyzes palmitate synthesis. Palmitate is a saturated fatty acid that serves as a key precursor for many lipid species, including those that constitute intracellular membranes. Multiple lipidomic studies demonstrate that human coronavirus infection profoundly alters cellular lipid profiles, with increased abundance of many specific lipid species [15,16]. To broadly assess lipid-associated membrane staining, we stained infected cells with a lipophilic fluorescent membrane dye. We observed increased staining intensity in A549 cells infected with HCoV-OC43, compared to mock uninfected controls (Figure 3A). We then quantified the lipid staining intensity on a per-cell basis, which demonstrated a significant increase with infection (Figure 3B,C).

We next tested the hypothesis that IRE1α contributes to the increased lipid-associated cellular staining during HCoV-OC43 infection. We found that the increased staining intensity in infected cells was reversed by the IRE1α inhibitor 4μ8C (Figure 3A–C), suggesting that IRE1α promotes lipid accumulation during infection. To determine whether IRE1α activation alone is sufficient to drive lipid accumulation, we treated cells with the small molecule IXA4, which specifically activates IRE1α, without affecting other branches of the unfolded protein response or other cellular stress responses [23]. We found that treatment with IXA4 increased lipid-associated cellular staining, and this was reversed by 4μ8C (Figure S3A,B), together indicating that IRE1α activation is sufficient to drive cellular lipid accumulation, independently of viral infection.

### 3.4. IRE1α Promotes HCoV-OC43 Infection via Fatty Acid Synthesis

**Figure 4:**
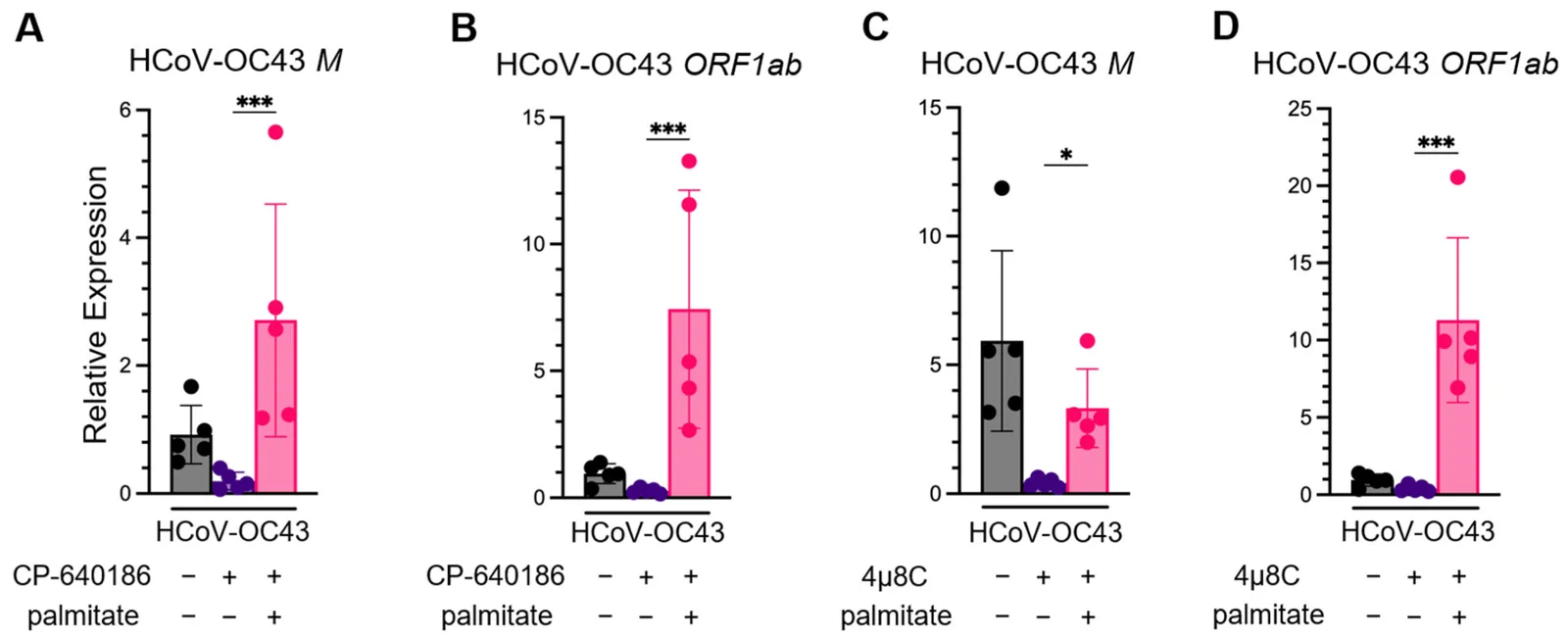
Exogenous palmitate restores viral infection in cells treated with an acetyl-CoA carboxylase (ACC) or IRE1α inhibitor. (**A**–**D**) Cells were pretreated with DMSO vehicle control, ACC inhibitor CP-640186 (**A** + **B**) or IRE1α inhibitor 4µ8C (**C** + **D**), then infected with HCoV-OC43 at an MOI of 0.01. At the time of infection, cells were treated with 100 µM BSA-conjugated palmitate or an equivalent concentration of BSA-conjugated control. RNA was harvested 48 h post-infection for quantitative RT-PCR analysis. Relative expression of HCoV-OC43 viral RNA was determined using two different primer pairs. Data are means +/− SD of five replicate wells of a cell culture plate and are representative of three independent experiments. * *p* < 0.05, *** *p* < 0.001 by one-way ANOVA.

Our results thus far demonstrate that IRE1α promotes increased cellular lipid content and that ACC activity, like IRE1α, is required for efficient HCoV-OC43 infection. We therefore hypothesized that bypassing the first steps in de novo lipogenesis might restore viral replication. Palmitate is the product of the combined activities of ACC and FASN, but exogenous BSA-conjugated palmitate can be taken up by cells to replace these enzymatic steps and provide the substrate for the subsequent synthesis of cellular lipid species [24,25,26,27]. We found that provision of exogenous BSA-conjugated palmitate rescued viral RNA replication in HCT-8 cells in the setting of ACC inhibition by CP-640186 (Figure 4A). To verify this result, we used a second primer pair to quantify viral RNA, which yielded similar results (Figure 4B).

We next tested the hypothesis that IRE1α promotes HCoV-OC43 infection by upregulating the first steps in de novo lipogenesis. Exogenous BSA-conjugated palmitate restored HCoV-OC43 infection in HCT-8 cells treated with the IRE1α inhibitor 4μ8C. This effect was confirmed by qRT-PCR analysis of viral RNA using two distinct primer sets (Figure 4B,C). Together, these results suggest that IRE1α-associated lipid metabolic changes may contribute to HCoV-OC43 infection.

## 4. Discussion

Coronaviruses are highly dependent on host cell processes to facilitate their replication, but the mechanisms by which these viruses manipulate host cells are incompletely understood. Based on previous findings that XBP1, downstream of IRE1α, is required for optimal infection [11,12], we hypothesized that the gene expression program mediated by this transcription factor contributes to creating a favorable cellular environment for viral replication. XBP1 has been demonstrated to promote de novo fatty acid synthesis, likely to facilitate ER expansion as a compensatory response to alleviate ER stress [28,29,30]. We observed that pharmacologic inhibition of ACC, the rate-limiting enzyme initiating de novo fatty acid synthesis, impaired viral replication, similar to previous findings with FASN inhibition [16,31]. We hypothesize that products downstream of ACC and FASN aid viral replication by increasing cellular lipid content, consistent with the increased lipophilic staining we observed in infected cells, and possibly reflecting intracellular membrane remodeling that shields viral nucleic acid synthesis from innate immune detection. These results underscore the importance of lipid metabolism for coronavirus replication, and our findings build upon this literature by suggesting IRE1α as a critical upstream regulator of these metabolic changes. Although multiple publications have independently concluded that various human coronaviruses require activity of IRE1α to support efficient viral replication [11,12,13,14], a conflicting study [32] utilized CRISPR/Cas9 gene editing to knock out IRE1α and XBP1 and found no detectable effect on HCoV-OC43 titer. Potential reasons for this discrepancy could include differences in the cell culture model systems, such as variability in fatty acid concentrations between different batches of fetal bovine serum [33], which could impact the need for IRE1α-mediated fatty acid synthesis. In addition, genetic deletion of IRE1α or XBP1 triggers compensatory activation of signaling and metabolic pathways [34,35,36], potentially attenuating detectable phenotypes.

Several limitations of the current study should be acknowledged. First, these experiments were performed using cultured cells and HCoV-OC43 as a model human coronavirus; additional studies in primary airway epithelial cultures, organoid systems, and animal models will be necessary to determine the physiologic relevance of these findings and applicability to other human coronaviruses, including SARS-CoV-2. Second, our results do not demonstrate whether XBP1 is directly responsible for the observed changes in lipogenic gene expression, and the involvement of XBP1 is inferred from IRE1α inhibition. Third, although our rescue experiments with exogenous palmitate strongly support a role for fatty acid synthesis downstream of IRE1α, the precise lipid species required for viral replication remain undefined. Coronaviruses induce complex remodeling of multiple lipid classes, and future lipidomic analyses will be important to identify the specific membrane components regulated by IRE1α signaling. In addition, IRE1α likely supports coronavirus infection through multiple mechanisms beyond lipogenesis, including regulation of viral entry factors and broader ER homeostasis pathways [11,12].

Despite the limitations of our study, these findings may have broader implications for understanding why metabolic diseases associated with ER stress increase susceptibility to severe coronavirus infection. Clinical studies from the COVID-19 pandemic identified advanced age, diabetes, obesity, hypertension, and chronic kidney disease as major risk factors for severe SARS-CoV-2 disease [2,3,4]. Many of these conditions are characterized by both chronic ER stress and dysregulated lipid metabolism [5,6,7]. Our results using HCoV-OC43 as a model raise the possibility that heightened basal activation of the IRE1α-XBP1 pathway in these settings could create a metabolically favorable environment for coronavirus replication. Furthermore, the ability of selective IRE1α inhibitors to suppress HCoV-OC43 replication without significant cytotoxicity [11] suggests that host-directed interventions may represent a potential antiviral strategy. Preclinical studies in other disease models indicate that IRE1α inhibitors are safe and well-tolerated, supporting the potential for clinical translation [37,38,39]. Dependence on host signaling and metabolic pathways may be less susceptible to rapid viral mutational escape than direct-acting antivirals, providing a possible therapeutic advantage. In summary, our study suggests that ER stress signaling interfaces with host metabolic remodeling during coronavirus infection and implicates lipogenesis as a potential downstream effector associated with IRE1α activity.

## Data Availability

The original contributions presented in this study are included in the article/Supplementary Material. Further inquiries can be directed to the corresponding author.

## Supplementary Materials

The following supporting information can be downloaded at https://www.mdpi.com/article/10.3390/v18090996/s1. Figure S1: Control RNA abundance is unaffected by infection or IRE1α inhibition; Figure S2: IRE1α promotes the transcription of enzymes involved in fatty acid synthesis; Figure S3: IRE1α-dependent increase in lipid stain intensity is independent of HCoV-OC43 infection.

## Abbreviations

The following abbreviations are used in this manuscript:

ACC	Acetyl-CoA carboxylase
ACE2	Angiotensin-Converting Enzyme 2
ANOVA	Analysis of Variance
BSA	Bovine serum albumin
cDNA	Complementary DNA
DMEM	Dulbecco’s modified Eagle’s medium
ER	Endoplasmic reticulum
FASN	Fatty acid synthase
FBS	Fetal bovine serum
HCoV-OC43	Human coronavirus OC43
IRE1α	Inositol-requiring enzyme 1 α
MOI	Multiplicity of infection
NIAID	National Institute of Allergy and Infectious Diseases
NIH	National Institutes of Health
qRT-PCR	Quantitative RT-PCR
SCD1	Stearoyl-CoA desaturase 1
SD	Standard deviation
