# Supplementary material for: IRE1α Is Associated with Host Lipid Biosynthesis During Human Coronavirus OC43 Infection
Source: Viruses. 2026 Sep 10;18(9):996. doi: 10.3390/v18090996 (PMC13611892; doi:10.3390/v18090996)
Supplement: Supplementary file 1 [file viruses-18-00996-s001.zip › viruses-4495745-supplementary.pdf]

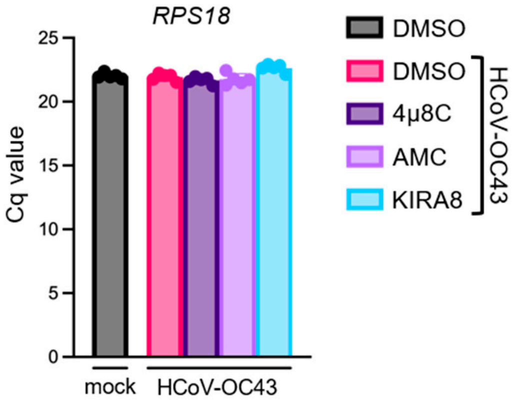

Figure S1. Control RNA abundance is unaffected by infection or IRE1α inhibition. Cells were pretreated with DMSO vehicle control, IRE1α nuclease inhibitor 4μ8C, inactive control AMC, or IRE1α kinase and nuclease inhibitor KIRA8. Treated cells were infected with HCoV-OC43 at an MOI of 0.01 and RNA was harvested 48 hours post-infection for quantitative RT-PCR analysis and the Cq values for the housekeeping gene *RPS18* are shown indicating similar control RNA abundance across conditions, consistent with absence of marked cytotoxicity. Data are means +/− SD from five replicate wells of a cell culture plate and are representative of three independent experiments.

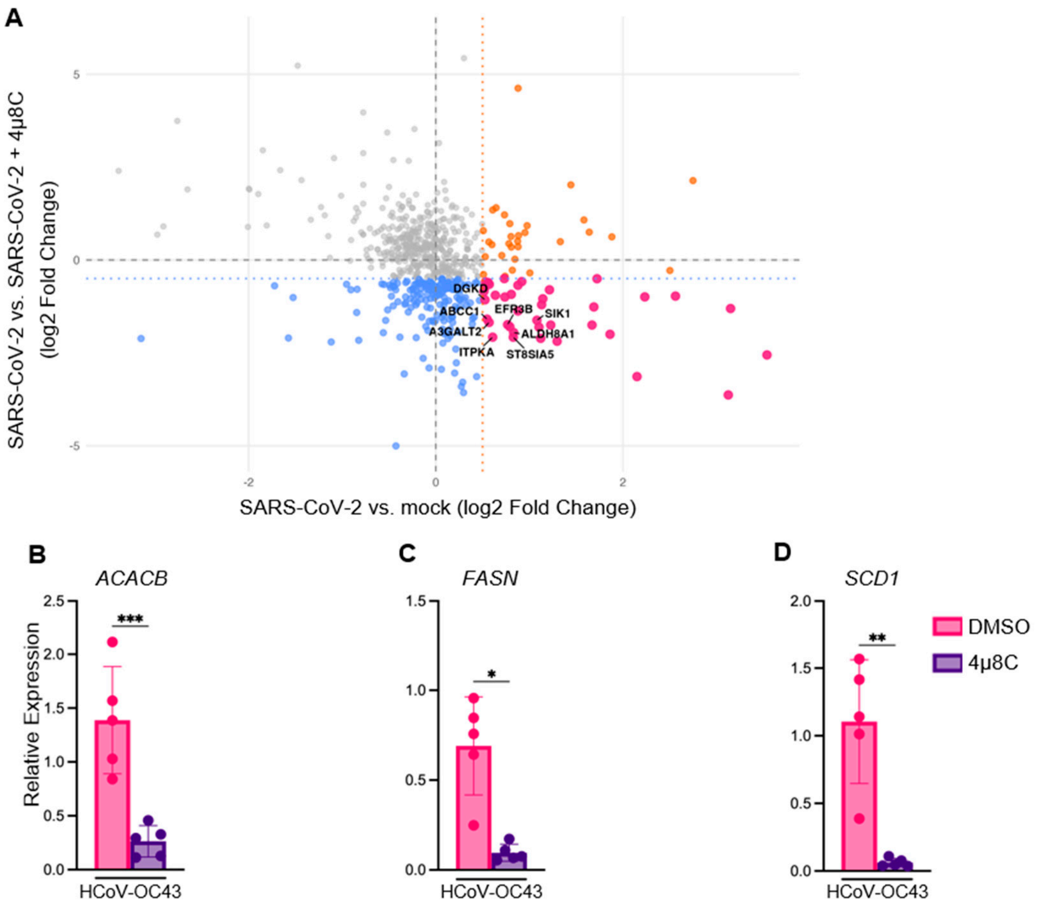

Figure S2. IRE1α promotes the transcription of enzymes involved in fatty acid synthesis. **(A)** Cells were infected with SARS-CoV-2 with or without IRE1α inhibitor 4μ8C. Total RNA was extracted 24 hours post-infection for RNA sequencing analysis. Differential expression of transcripts were calculated and log-transformed. Transcripts relating to lipogenesis, as defined by multiple software packages, were plotted. Expression of transcripts in mock vs. SARS-CoV-2 infected cells is shown along the x-axis. Expression of transcripts in SARS-CoV-2 infected cells with vs. without 4μ8C treatment is shown along the y-axis. Data are representative of two independent biological experiments. Threshold lines at ± 0.5 log2 fold change are shown as dotted lines. **(B-D)** Cells were pretreated with DMSO vehicle control or IRE1α inhibitor 4μ8C. Treated cells were infected with HCOV-OC43 at an MOI of 0.01 and RNA was harvested 48 hours post-infection for quantitative RT-PCR analysis. Relative expression was determined for *ACACB* **(B)**, *FASN* **(C)**, and *SCD1* **(D)**. Data are means +/− SD from five replicates and are representative of three independent experiments. * $p < 0.05$, ** $p < 0.01$, *** $p < 0.001$ by one-way ANOVA.

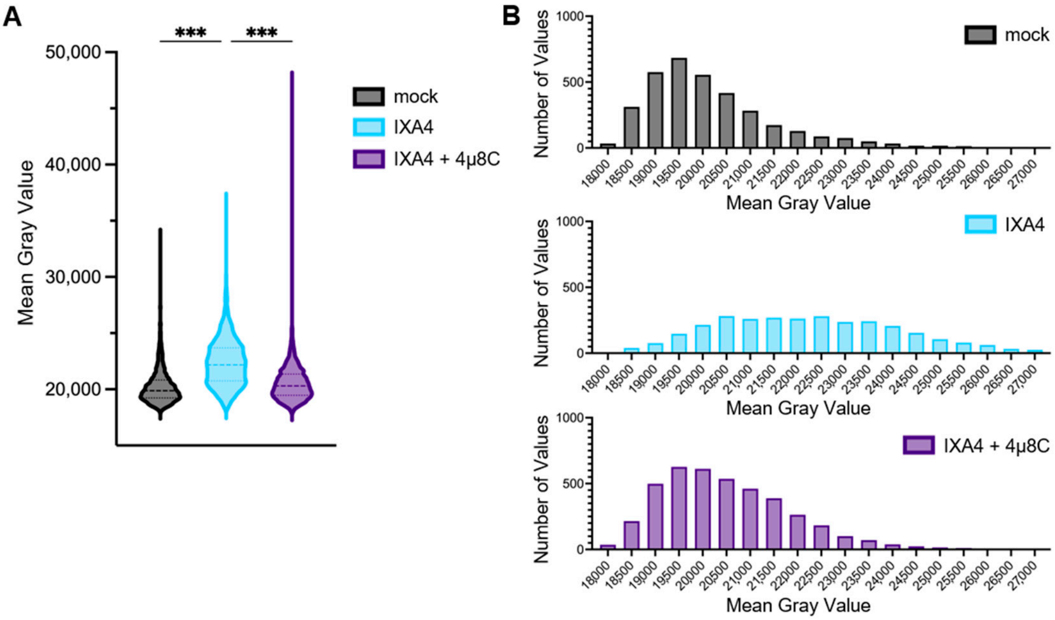

Figure S3. IRE1α-dependent increase in lipid stain intensity is independent of HCoV-OC43 infection. **(A+B)** Cells were pretreated with DMSO vehicle control or IRE1α inhibitor 4µ8C, then treated with selective IRE1α activator IXA4 for 24 hours. Staining was performed on fixed cells for polar. Quantification of the fluorescence intensity of each cell was computed as a Mean Gray Value. **(A)** The violin plot shows the Mean Gray Value for each cell captured in twelve total ROIs across two wells in one experiment. **(B)** Histograms show the distribution of the Mean Gray Values for each condition. Data are representative of two independent experiments. *** $p < 0.001$ by one-way ANOVA.
